# Yemen Advanced Field Epidemiology Training Program: An Impact Evaluation, 2021

**DOI:** 10.3390/epidemiologia4030024

**Published:** 2023-06-23

**Authors:** Maeen Abduljalil, Abdulhakeem Al Kohlani, Aisha Jumaan, Abdulwahed Al Serouri

**Affiliations:** Yemen Field Epidemiology Training Program, Ministry of Public Health and Population, Sana’a 72738, Yemen

**Keywords:** Yemen, advanced, field, epidemiology, training, program, outcomes, evaluation

## Abstract

This is the first evaluation of the Yemen Field Epidemiology Training Program (Y-FETP) to assess if it met its objectives. We collected data using mixed methods including desk review, a focus group discussion with the Y-FETP staff, in-depth interviews with 21 program stakeholders, and an online survey for the program’s graduates. We transcribed/analyzed qualitative data using explanatory quotations and survey data using descriptive methods. The desk review indicated that Y-FETP covers 18 (82%) out of 22 governorates and conducted >171 outbreak investigations, 138 surveillance system analyses/evaluations, 53 planned studies, published >50 articles and had >155 accepted conference abstracts. Qualitative findings showed Y-FETP helped save lives and reduced morbidity/mortality using building capacities in outbreak response; provided evidence-based data for decision-making; and increased awareness about public health issues. An online survey showed that Y-FETP helped 60 to 80% of graduates conduct outbreak investigations, surveillance analysis/evaluation, manage surveillance systems/projects, engage in public health communication (reports/presentation), and use basic statistical methods. However, the evaluation revealed that Y-FETP is primarily funded by donors; thus, it is not sustainable. Other challenges include low graduate retention and limited training in policy development and management. Y-FETP achieved its main objectives of increasing the number of epidemiologists in the workforce, making a positive impact on public health outcomes.

## 1. Introduction

The Yemen Field Epidemiology Training Program (Y-FETP) was established in 2011 amidst political instability followed by an ongoing war that has lasted for eight years; however, it has not been evaluated to date. Evaluation of training programs is important to assess if the programs achieve their objectives, to identify weaknesses and strengths, and make improvements for future training. Therefore, many of the FETP programs have conducted evaluation of their programs and shared their learnt experiences and lessons [1,2,3,4,5,6,7,8,9,10]. These studies revealed that FETPs contributed to the improvement of a skilled workforce in field epidemiology, but additional efforts are needed to scale up the programs and ensure their sustainability. Nevertheless, the vast majority of published FETP evaluations concentrate on process indicators and do not assess mid- or long-term impact. The Yemen Field Epidemiology Training Program (Y-FETP) is a 2-year training program utilizing a “learning while doing” approach. It was established to address the lack of skilled public health workers, especially epidemiologists, and to support the Ministry of Public Health and Population (MoPHP), in meeting the Global Health Security Agenda (GHSA) target of having one trained field epidemiologist per 200,000 members of the population by 2025 [2]. The Y-FETP has three main objectives: build national epidemiological capacities to respond to outbreaks, thus reducing morbidity and mortality; provide decision-makers with evidence-based data; and increase awareness about public health issues using published scientific articles and conference abstracts.

The program started with an advanced two-year tier in 2011, followed by a three-month frontline tier in 2017, and a nine-month intermediate tier in 2022. The program has graduated five advanced cohorts (53 trainees) and 19 frontline cohorts (412 trainees).

The Y-FETP is housed at the Disease Control and Surveillance General Directorate under the Primary Health sector of MoPHP. The program employs a director who is supported by a technical advisor, three epidemiologists and two administrative staff. The program was initially financially supported by the U.S. Center for Disease Control and Prevention (CDC) and by the Training Program in Epidemiology and Public Health Interventions Network (TEPHINET) since 2015, with ad hoc support from the Eastern Mediterranean Public Health Network (EMPHNET) and World Health Organization (WHO). Although previous reports on the Y-FETP described how it contributed to the development of a skilled work force and improved response to outbreaks such as cholera and COVID-19 [2,11,12], they did not consider input from the different Y-FETP stakeholders. We performed this evaluation in 2021 to assess whether the advanced Y-FETP achieved its objectives, and to use the results to improve the program. Findings will help develop high-quality training and assure the program’s effectiveness in improving public health in Yemen [2].

## 2. Materials and Methods

### 2.1. Study Design and Site

This evaluation is descriptive and follows Kirkpatrick’s model for evaluating the results of training programs. It assesses formal and informal training methods using four levels of criteria: reaction, learning, behavior, and results [13,14]. It focusses on levels 3 (behavior) and 4 (results) that measure the degree to which trainees apply the learnt material, and the direct results; targeted outcomes are realized. 

### 2.2. Sampling and Sample Size

The program had a list that included names and contact details of the following Y-FETP stakeholders: (i) 5 Y-FETP technical staff, (ii) 5 senior MoPHP policymakers who oversee the Y-FETP, (iii) 17 Program directors who hosted the Y-FETP residents, (iv) 4 organizations that were employing the Y-FETP graduates, and (v) the 43 Y-FETP graduates from the date of its establishment to February 2020.

### 2.3. Collection Methods and Tools

We collected the data between August and October 2021 using mixed quantitative and qualitative methods that had been used in previous global and regional FETPs evaluations [1,15,16]. These tools include a desk review to extract information about the Y-FETP and its activities; focus group discussions (FGDs) with Y-FETP staff; individual in-depth interviews (IDIs) with senior MoPHP policy makers, program directors who hosted Y-FETP residents or graduates, and organizations that employed them; and an online survey for Y-FETP graduates. 

### 2.4. Data Analysis

We analyzed quantitative data using descriptive methods (e.g., frequencies, percentage, etc.). We transcribed/analyzed qualitative data using explanatory quotations. We presented quotes that revealed the strengths and weakness of the program or conveyed a particular experience or statement. 

## 3. Results

The response rates were 100% for the FGD with Y-FETP technical staff, 94% for IDIs with program directors, 100% for senior policymakers, and 50% for organizations that employed graduates. The response rate for the online survey of graduates was 30/43 graduates (70%). Table 1 shows the demographic characteristics of the online survey respondents. Seventy percent were males, 47% were 40–45 years of age, around 50% had a master’s degree, and 30% were physicians working onsite. The graduates were nominated from 11 governorates, 50% from Amanat Al Asimah (Sana’a city), and about 66% being currently employed by the MoPHP.

All FGDs and the majority of IDI participants indicated that Y-FETP helped to identify public heath challenges through performing many epidemiologic activities, and they provided useful recommendations to the programs as well as MoPHP decision-makers to overcome such challenges. Most participants agreed that Y-FETP residents and graduates have good skills and are superior to peers in other training programs, e.g., the Master of Public Health and Community Medicine Diploma. They stressed that such skills are more needed during the current siege and war situation that has made it difficult to bring external consultants to Yemen. 

“We benefited from the accumulated experiences of the CDC and other FETPs to build the epidemiological capacity of our residents. Thus, the curriculum ensures the best mix between theoretical and practical components that a filed epidemiologist requires”. **Y-FETP staff**.

“Y-FETP residents supported different MoPHP programs through performing a wide-range of epidemiological activities such as analyzing and evaluating surveillance data; and conducting filed investigations. Thro ugh such activities not only epidemiological capacities of the residents were strengthened but also the capacities of the programs and the staff”. **Senior MoPHP Policymaker**

“During the 2005 polio outbreak in Yemen, there was a shortage of local epidemiologist, and investigation was not done in a proper and timely manner … the reports were also weak and incomplete; therefore the response was inadequate and external expertise was needed … But after we had Y-FETP when the cholera outbreak occurred in 2017, we noticed marked improvement in the quality of the investigations reports and we were able to convey scientific and evidence-based messages regarding the situation, both to national and the international community …. Moreover, the response was mainly led by local staff”. **Organization staff employee**.

“Y-FETP graduates had robust scientific background and good communication skills … This came from the curriculum which focuses on theoretical and practical approach. So, the residents and graduates were well versed with field and practice compared to many other teaching programs”. **Program director**.

“In the past, international WHO consultants routinely came to evaluate the Acute Flaccid Paralysis (AFP) program thrice a year …. these evaluations were interrupted with the war and blockade in the country as there was no possibility to recruit international consultant to do the evaluation …. Thankfully, the Y-FETP residents played a major role towards this end and conducted and published two evaluations for the AFP program and helped us identify gaps and correct deviations on a timely manner”. **AFP Program Director**.

**Figure 1:**
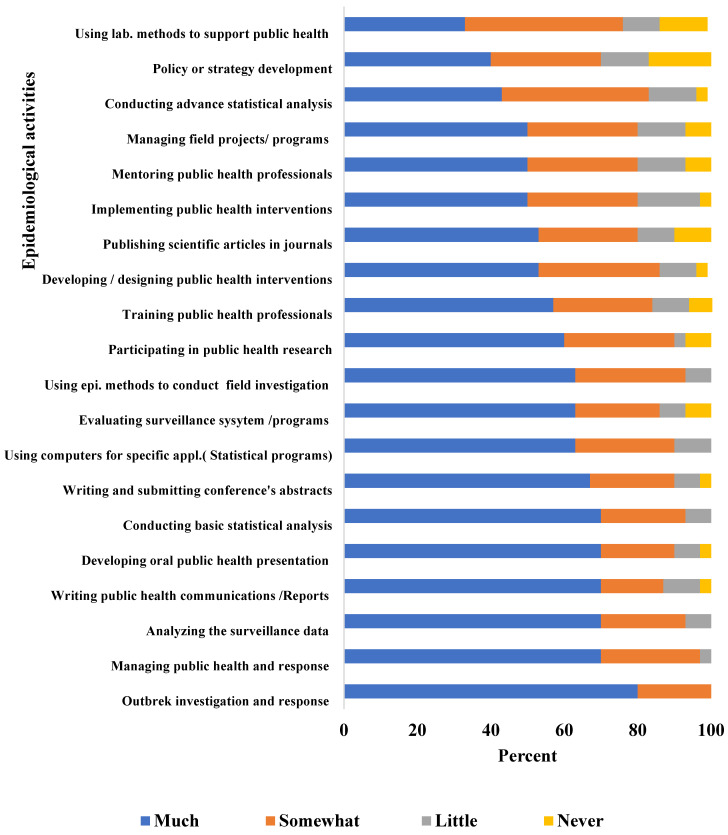
The degree to which the program helped the graduates to perform epidemiologic activities, Yemen Field Epidemiology Training Program Graduates’ Online Survey, Yemen, 2021.

Such qualitative findings support the quantitative results obtained from the graduates’ online survey. Figure 1 shows that 60–80% of the graduates reported that Y-FETP greatly helped them in performing epidemiological field activities such as outbreak investigation and response, surveillance analysis/evaluation, managing surveillance systems/projects, public health communication (reports/presentation), and applying basic statistical methods. 

**Figure 2:**
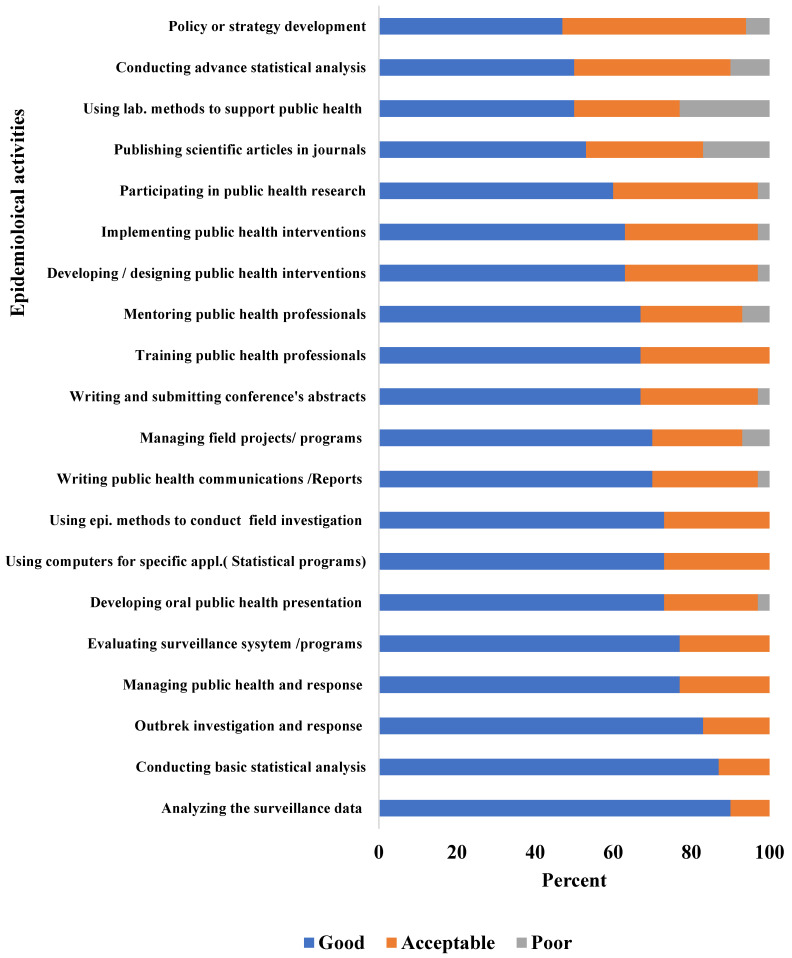
Graduates’ self-evaluation of their skills in performing epidemiologic activities, Yemen Field Epidemiology Training Program Graduates’ Online Survey, Yemen, 2021.

In addition, 70% to 90% of graduates rated their skills as good in performing epidemiological field activities such as outbreak investigations, surveillance analysis/evaluation, managing surveillance systems/projects, public health communication (reports/presentation), using computers for specific applications relevant to public health practice, using epidemiological methods to design/conduct studies or field investigations, managing field projects/programs and conducting basic statistical analysis (Figure 2). 

The desk review results showed that Y-FETP residents and graduates conducted >171 outbreak investigations, 138 surveillance system analyses/evaluations, and 53 planned studies. According to IDI with one of the policy makers, these numbers are remarkable. 

“Before Y-FETP, the MoPHP usually conducted 2–3 outbreak investigations on ad hoc bases and according to availability of funds from the Ministry of Finance … In many situations there was no funds for outbreak investigation … the untrained governorate surveillance officers investigated some of these outbreaks …. furthermore, no surveillance systems were analyzed nor evaluated before starting the Y-FETP…Only WHO did one external evaluation for acute flaccid surveillance system ……………. Also, the General Directorate for Diseases Control and Surveillance was not able to conduct planned studies to investigate health problem”. **Senior MoPHP policymaker**.

Qualitative and quantitative findings showed that the Y-FETP helped deliver evidence-based data that supported decision making using analysis and evaluation of surveillance programs, filing investigations of the priority health problems, and timely outbreak investigation and control, thus leading to a faster, more efficient response, reducing both morbidity and mortality and helping save lives. FGDs with Y-FETP staff and IDIs with senior policymakers, program directors, and employer organization staff were recorded to recount examples. 

“One of the 2nd cohort residents who evaluated the electronic integrated Diseases Early Weaning System (eIDEWS) program showed that one of the reasons for weak reporting was due to using none SMART phones that cannot support some of the reporting functions. Therefore, she recommended that the program provide the field coordinators with SMART phones …. The eIDEWS program was able to implement the recommendation …. After that reporting improved considerably that improved timely alerts and response to outbreaks”. **Y-FETP staff**.

“The residents and graduates’ analyses and filed investigations contributed remarkably to making better decisions, solving problems and making policies”. **Senior MoPHP policymaker**.

“The Y-FETP residents analyzed surveillance data and found that the incidence of some diseases increased in some areas … They highlighted the problem and presented it to the decision-makers in the MoPHP and stakeholders such as WHO …. The MoPHP with support from WHO in turn found solutions and overcome the problem”. **Organization staff employee**.

“Y-FETP team went to investigate a measles outbreak …. The team found that 95% of the affected children were unvaccinated therefore, the team recommend to conduct an immunization campaign…. Thus UNICEF agreed to conduct such campaign …. We noticed marked reduction of measles cases after that”. **Y-FETP staff**.

“In Expand Program for Immunization, the Y-FETP contributed remarkably in providing data on the spread of vaccine preventable diseases and outbreaks and identified areas of low vaccine coverage, this helped us in getting funds to conduct vaccination campaign and outreach activities” **EPI Program Director.**

Findings from the graduates’ online survey showed that 97% of the Y-FETP graduates participated in investigation and control of important outbreaks such as COVID-19, cholera, and diphtheria. 

**Figure 3:**
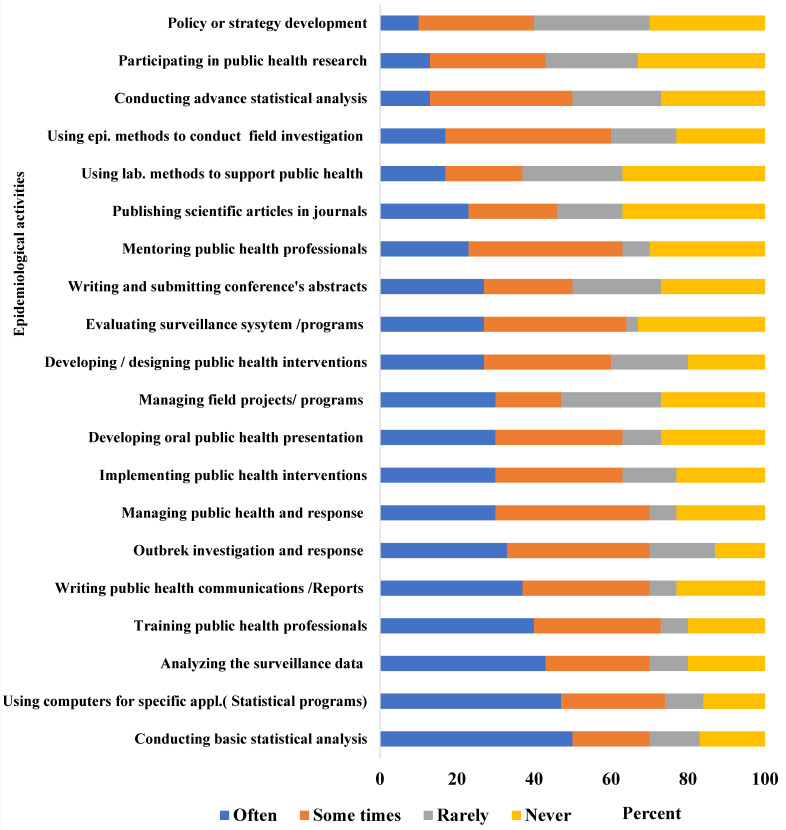
The degree of engagement of graduates in epidemiologic activities, Yemen Field Epidemiology Training Program Graduates’ Online Survey, Yemen, 2021.

Furthermore, through engagement with wide range of epidemiological activities such as outbreak investigation and analyzing and evaluating surveillance systems/programs, these activities resulted in a better understanding of the current status of the health system and the challenges it is facing, especially under the difficult circumstances in Yemen; this contributed to evidence-based decisions being made (Figure 3).

Finally, all participants of the FGDs and IDIs indicated that Y-FETP had a significant role in increasing the awareness of health issues through publishing in peer-reviewed journals as well as through presenting at national, regional, and international conferences. 

“Y-FETP had a significant role in documenting health problems through publishing in scientific journals and participation in national, regional and international conferences”. **Y-FETP Staff.**

“The Y-FETP residents and graduates analyzed programs’ surveillance data such as acute flaccid paralysis, measles, and other programs. The outcome of the analyses was shared in international conferences and published in peer-reviewed journals”. **Senior MoPHP policymaker**.

“A team from our program participated with Y-FETP on a scientific research on HIV/AIDS situation in Yemen that was presented in a conference in Thailand”. **Program Director**.

In fact, the desk review showed that Y-FETP published >50 scientific articles in peer-reviewed journals and presented >155 abstracts at national, regional, and international conferences. According to the IDIs, publication rarely occurred before the program was implemented: 

“Before Y-FETP, the general directorate for diseases control and surveillance published only three papers in collaboration with WHO and CDC”. **Senior MoPHP policymaker**.

Findings from the graduates’ online survey showed that around two-thirds of Y-FETP graduates published one or more articles in scientific peer-reviewed journals, and all had submitted accepted abstracts in national, regional, and international conferences, e.g., TEPHENT global conferences, EMPHNET regional conferences and Epidemic Intelligence Service (EIS) conferences. 

Despite the many remarkable achievements of the program, it is threatened by several factors. One of the Y-FETP staff who participated in the FGD indicated that one of these challenges is its dependence on international funding (TEPHNIET), making it less likely to be sustainable. 

“The program mainly depends on TEPHINET financial support e.g., for outbreak investigation, staff salaries, residents’ stipend, running cost etc”. **Y-FETP staff**.

The desk review identified several challenges. The first is that the program only covers 18 (82%) out of the 22 governorates in Yemen, and half of the trainees who joined the program came from the capital city Sana’a, i.e., Amanat Al Asimah. This was also raised during the Y-FETP FGD. 

“We, as a national program, always invite and encourage all Yemeni governorates to nominate their eligible candidates to enroll in the program… some governorates did not respond for different reasons e.g., no eligible or interested candidates, or their candidates did not pass the screening course”. **Y-FETP Staff**

The second challenge is low retention of graduates by the National Health System (NHS). The desk review showed that out of the 43 graduates, 19 (44%) were currently working with donor organizations, e.g., WHO, UNICEF, MSF, Save the Children. A policymaker raised this challenge in one of the IDIs: 

“Due to the current war and blockade on Yemen, many of the graduates prefer to join donor organization for better salary and living condition”. **Senior MoPHP policymaker**.

Finally, most of senior policymakers and program directors pointed out that Y-FETP residents and graduates do not contribute directly to policies and strategies and have weak management skills. 

“… despite strengths, residents and graduates do not play a strong role in supporting the ministry and programs in developing policies and strategies and many are weak in management skills”. **Senior MoPHP Policymaker**

Graduates raised similar limitations in their responses to the online survey: only 40% reported that Y-FETP helped them in policy development (Figure 1) and 47% rated their skills as good (Figure 2). Furthermore, less than 10% of graduates reported that they often engaged in policy development (Figure 3) and only 30% reported being regularly engaged in program management.

## 4. Discussion

An important indicator for achieving a training program’s—such as the Y-FETP—intended outcomes and impact the dependence on the graduate’s ability to apply those learned skills at work to support and improve the health system, where the Y-FETP plays a major role in an enriched NHS, as indicated by several evidence-based data. For example, a timely response to an outbreak involves determining the cause of the outbreak, and the population at risk, to allow for effective implementation of control measures, and thus reduce morbidity and mortality. Both the qualitative and quantitative findings showed that the Y-FETP program helped build national epidemiological capacities to respond to outbreaks consistent with the International Health Regulation and GHSA [17]. Furthermore, the results showed that Y-FETP played a critical role in building national epidemiological capacities through providing residents with theoretical and practical skills. This finding is similar to results from the Central America Field Epidemiology Training Program’s evaluation, which showed that the FETP helped in the development of high-caliber field epidemiologists at various levels of the public health system [18]. Similarly, evaluation of the United Kingdom’s FETP found a direct impact on capacity building through training fellows and an indirect impact through developing other staff within local teams and bringing insights from other areas [15]. 

Our results confirmed the findings from the Indian Ocean FETP evaluation that specified the need for competency-based field training programs to build capacity to support the health system [6]. This was accomplished through the many epidemiological activities the residents performed, including analyzing and evaluating surveillance system data, outbreak investigation and field studies. 

War and the blockade on Yemen disrupted the NHS with the severe shortage of resources and difficulty in bringing international expertise, especially from International agencies. A study in the Eastern Mediterranean region (EMR) showed that the FETPs played a vital role in the region as many countries experienced unrest as a result of wars, political conflicts, massive forced displacement and disease outbreaks [1]. Our results from the quantitative data supported the EMR study and showed that the Y-FETP built the capacities of its graduates in key field epidemiological activities such as outbreak investigations, surveillance analysis/evaluation, public health communication (reports/presentation), and basic statistical analysis. These important skills were instrumental in responding to the challenges facing NHS such as cholera, diphtheria, and COVID-19 [1]. 

Tacker and Buffington defined applied epidemiology as “transforming findings to policy and action” [19]. The cornerstone in establishing the Y-FETP was to transfer the results of the various activities to evidence-based actions. This evaluation clearly demonstrated that Y-FETP met its objective through utilizing the results of the many epidemiological field activities for evidence-based decisions. There was consensus among all participants that the program played a crucial role in helping MoPHP decision-makers, program directors, and stakeholders to make informed decisions and overcome shortcomings. Such findings are consistent with similar evaluations of FETPs [1,16,17]. 

Sharing findings from scientific research and field investigation is an important tool for increasing awareness about the health problems and challenges facing the NHS. Being part of the EMPHNET Regional Field Epidemiology Network and the TEPHINET International Field Epidemiology Network, Y-FETP shared knowledge and increased awareness regarding the most challenging global public health problems and threats. The desk review and qualitative and quantitative findings showed that Y-FETP achieved this using published scientific articles in peer-reviewed journals and abstracts that were presented in national, regional, and international conferences. Y-FETP contributed to a better understanding and awareness of Yemen NHS problems and challenges and exposed emerging and reemerging disease threats in Yemen such as COVID-19, diphtheria, and cholera and their containment measures. [11,12,20,21,22,23,24,25]. 

This evaluation identified some gaps that need attention. First, this evaluation revealed that the Y-FETP depends solely on donor support, which is a major challenge for the survival of the program [26]. Evaluation of the Ghana Field Epidemiology and Laboratory Training Program showed that over the years, the donors, stakeholders, and residents funded the advanced FETP program while the WHO fully funded the frontline FETP program [26]. A global evaluation found that, of the 19 programs established during 1980–2000 with CDC support, 17 continue to function, graduate trainees, and provide service [17]. For sustainability purposes and long-term contribution of the FETPs toward strengthening public health, the studies suggested that FETP programs should be attached to the ministries of health or other public health institutions with gradual replacement of the financial and technical support received from external donors and partners [27]. Therefore, sustainability of Y-FETP requires proper attention from the national health system, exploring different stakeholders and potentially support from graduates. Gradual replacement of donor funds using governmental funds may be difficult in the current situation of war and blockade in Yemen; however, the Y-FETP should consider piloting some innovative approaches such as partnership, introduction of self-sponsorship, and service provision/consultancies. 

Furthermore, the Y-FETP needs to widen its national coverage and target all governorates using more coordinating and sensitizing visits to the non-represented governorates to discuss obstacles and overcome possible challenges.

Migration of health professionals from the public health system is a major concern that hinders development in Yemen [28]. The Y-FETP recommends that the MoPHP employs Y-FETP graduates at the national level of MoPHP and its governorate branches as field epidemiologists or managers of national disease prevention and control programs, and in leadership positions requiring a high degree of analytic capability. Currently, more than 40% of Y-FETP graduates leave the NHS to work with international organizations. Such a departure is usually for better salary or living conditions. Other factors include better working conditions, professional supervision and management, and greater access to education and training opportunities. Therefore, improving public health salaries and working conditions would be useful measures to improve retention.

Since only a small percentage of Y-FETP graduates became engaged in policy development and program management, similar to published results from global and EMR FETP evaluations [2,9], the Y-FETP should revisit its curriculum and include these topics.

Finally, this evaluation has strengths. First, it focused on level 3 and 4 of the Kirkpatrick model [12], i.e., “the degree of applying what was learned” and “the degree to which outcomes occur as a result of the training”, which intend to measure impact. Other strengths include collecting information from different sources (i.e., Y-FETP staff, senior MoPHP policymakers, program directors, and organization staff employees and graduates), and using different tools (i.e., FGD, IDIs, and an online survey) that allowed for triangulation and provided robust findings. Furthermore, because the graduate survey was anonymous and online, this likely decreased social desirability bias.

This evaluation also had some limitations. First, one of the Y-FETP residents conducted this internal evaluation; therefore, subjectivity could be a problem. Second, as the graduates’ capacities were self-assessed using an online survey, there may be an overrating. Thirdly, the evaluation elicited views and opinions from teams already involved in the program and from the program’s stakeholders that may be biased towards more favorable views, but there is no choice because the Y-FETP chooses the programs that it is more involved with, but the Y-FETP needs to coordinate with programs outside of the MoPH, such as climatic, environmental, cultural, and others, to achieve a unified health approach. Finally, this evaluation did not state longer-term outcomes and impacts such as changes, e.g., in morbidity and mortality, brought to the health system. These features will require a longer-term perspective, maybe in the situation of an external evaluation.

## 5. Conclusions

In conclusion, the Y-FETP achieved its objectives through building national epidemiologic capacities, providing decision-makers with evidence-based data, and increasing awareness about public health issues. However, it should consider ways to ensure sustainability through partnership, self-sponsorship, and service-provision. Furthermore, improving public health salaries and working conditions would be useful in improving graduate retention. Finally, the Y-FETP should work towards widening its national coverage and revisit its curriculum to strengthen residents’ capacities in policy development and managerial skills.

## Figures and Tables

**Table 1:** The demographic characteristics of the responding graduates, Yemen Field Epidemiology Training Program Graduates’ Online Survey, Yemen, 2021 (*n* = 30).

Variable	No.	%
Gender		
Males	21	70
Females	9	30
Age (years)		
<40	9	30
40–45	14	47
>45	7	23
Highest educational degree earned		
Master	16	54
Bachelor	10	33
Diploma	2	7
Part one community medicine	1	3
PHD	1	3
Position at working site		
Physician	9	30
Surveillance officer	8	27
Laboratorian	5	17
Program manager/director	4	13
Others	4	13
From which governorate the graduates were nominated		
Amanat Al Asimah	15	50
Hadramout	4	13
Abyan	2	7
Amarn	2	7
Other governorates	7	23
Current Employing agency		
Ministry of Public Health and Population	19	64
WHO	5	17
University	2	7
NGO	3	9
No job	1	3

## Data Availability

Not applicable.
